# Supplementary material for: Red meat consumption and its association with hypertension and hyperlipidaemia among adult Maasai pastoralists of Ngorongoro Conservation Area, Tanzania
Source: PLoS One. 2020 Jun 1;15(6):e0233777. doi: 10.1371/journal.pone.0233777 (PMC7263614; doi:10.1371/journal.pone.0233777)
Supplement: S1 File — (DOCX) [file pone.0233777.s001.docx]

## English Questionnaire

This is the modified questionnaire from WHO STEPS approach to NCD surveillance.

| **Survey Information** | | | | | | | | | | | | | | |
| --- | --- | --- | --- | --- | --- | --- | --- | --- | --- | --- | --- | --- | --- | --- |
| **Location and Date** | | | | **Response** | | | | | | | | | **Code** | |
| Centre/Village ID | | | | **└─┴─┘** | | | | | | | | | I1 | |
| Centre/Village name | | | |  | | | | | | | | | I2 | |
| Interviewer ID | | | | └─┴─┴─┴─┘ | | | | | | | | | I3 | |
| Date of completion of the instrument | | | | └─┴─┘ └─┴─┘ └─┴─┴─┴─┘  dd mm year | | | | | | | | | I4 | |
| **Consent, Interview Language and Name** | | | | **Response** | | | | | | | | | **Code** | |
| Consent has been read and obtained | | | | Yes | | | 1 | | | | | | I5 | |
|  |  |  |  | No | | | 2 **If NO, END** | | | | | |  |  |
| Interview Language | | | | Swahili | | | 1 | | | | | | I6 | |
|  |  |  |  | Maasai | | | 2 | | | | | |  |  |
|  |  |  |  |  | | |  | | | | | |  |  |
| Time of interview  (24-hour clock) | | | | └─┴─┘: └─┴─┘  hrs mins | | | | | | | | | I7 | |
| **Demographic Information** | | | | | | | | | | | | | | |
| **Demographic Information** | | | | | | | | | | | | | | |
| **Question** | | **Response** | | | | | | | | | | **Code** | | |
| Sex (*Record Male / Female as observed)* | | Male | | | | | | | 1 | | | C1 | | |
|  |  | Female | | | | | | | 2 | | |  |  |  |
| What is your date of birth?  *Don't Know 77 77 7777* | | └─┴─┘ └─┴─┘ └─┴─┴─┴─┘  dd mm year | | | | | | | | | | C2 | | |
| How old are you? | | Years | | | | | | | └─┴─┘ | | | C3 | | |
| What is the **highest level of education** you have completed? | No formal schooling | | | | | | | | 1 | | | C4 | | |
|  | Primary school completed | | | | | | | | 2 | | |  |  |  |
|  | Secondary school completed | | | | | | | | 3 | | |  |  |  |
|  | Completed High school or above | | | | | | | | 4 | | |  |  |  |
| What is your **marital status**? | Single/Never married | | | | | | | | 1 | | | C5 | | |
|  | Currently Married/Cohabiting | | | | | | | | 2 | | |  |  |  |
|  | Separated/Divorced. Widowed | | | | | | | | 3 | | |  |  |  |
| Which of the following best describes your **main** **work** status over the past 12 months? | No Job/student | | | | | | | | 1 | | | C6 | | |
|  | Pastoralist  Farmer | | | | | | | | 2  3 | | |  |  |  |
|  | Business | | | | | | | | 4 | | |  |  |  |
|  | Professional employed | | | | | | | | 5 | | |  |  |  |
| How many people older than 18 years, including yourself, live in your household? | Number of people | | | | | | | | └─┴─┘ | | | C7 | | |
| Taking **the past year**, can you tell me what the average earnings of the household have been?  *(RECORD ONLY ONE, NOT ALL 3)* | Per week | | | | └─┴─┴─┴─┴─┴─┴─┘ *Go to T1* | | | | | | | C8a | | |
|  | OR per month | | | | └─┴─┴─┴─┴─┴─┴─┘  *Go to T1* | | | | | | | C8b | | |
|  | OR per year | | | | └─┴─┴─┴─┴─┴─┴─┘ *Go to T1* | | | | | | | C8c | | |
|  | Refused | | | | 88 └─┴─┘ | | | | | | | C8d | | |
| **Step 1 Behavioral Measurements** | | | | | | | | | | | | | | |
| **Tobacco Use** | | | | | | | | | | | | | | |
| *Now I am going to ask you some questions about tobacco use.* | | | | | | | | | | | | | | |
| **Question** | **Response** | | | | | | | | | | | | **Code** | |
| Do you **currently** smoke any **tobacco** products, such as cigarettes, cigars or pipes? | Yes | | | | | 1 | | | | | | | T1 | |
|  | No | | | | | 2 *If No, go to A1* | | | | | | |  |  |
| Do you currently smoke tobacco products **daily**? | Yes | | | | | 1 | | | | | | | T2 | |
|  | No | | | | | 2 | | | | | | |  |  |
| How old were you when you **first started** smoking? | Age (years) | | | | | **└─┴─┘** | | | | | | | T3 | |
|  | Don’t know 77 | | | | |  |  |  |  |  |  |  |  |  |
| **Alcohol Consumption** | | | | | | | | | | | | | | |
| *The next questions ask about the consumption of alcohol.* | | | | | | | | | | | | | | |
| **Question** | | | **Response** | | | | | | | | | | **Code** | |
| Do you currently consume any alcohol such as beer, wine, spirits, homebrewed alcohol or any alcohol brought over the border/from another country? | | | Yes | | | | | 1 | | | | | A1 | |
|  |  |  | No | | | | | 2 | | | | |  |  |
| Have you consumed any alcohol within l2 **months**? | | | Yes | | | | | 1  *If Yes, go to A4* | | | | | A2 | |
|  |  |  | No | | | | | 2 | | | | |  |  |
| Have you stopped drinking due to health reasons, such as a negative impact on your health or on the advice of your doctor or other health worker? | | | Yes | | | | | 1 *If Yes, go to D1* | | | | | A3 | |
|  |  |  | No | | | | | 2 *If No, go to D1* | | | | |  |  |
| During the past 12 months, **how frequently** have you had at least one standard alcoholic drink? | | | Daily | | | | | 1 | | | | | A4 | |
|  |  |  | 5-6 days per week | | | | | 2 | | | | |  |  |
|  |  |  | 3-4 days per week | | | | | 3 | | | | |  |  |
|  |  |  | 1-2 days per week | | | | | 4 | | | | |  |  |
|  |  |  | 1-3 days per month  Less than one a month  Never | | | | | 5  6  7 | | | | |  | |
| Have consumed any alcohol within the past 30 days | | | Yes  No | | | | | 1  2 *If No, go to A13* | | | | | A5 | |
| During the **past 7 days**, did you consume any **homebrewed** alcohol? (Such as mbege) | | | Yes  No | | | | | 1  2 *If No, go to A13* | | | | | A6 | |
| **Diet** | | | | | | | | | | | | | | |
| *The next questions ask about the fruits, vegetables, salt and red meat that you usually eat. As you answer these questions please think of a typical week in the last year.* | | | | | | | | | | | | | | |
| **Question** | | | | **Response** | | | | | | | | | | **Code** |
| In a typical week, on how many days do you **eat fruit**? | | | | Number of days Don't Know 77 | | | | | | *└─┴─┘ If 0 days, go to D3* | | | | D1 |
| How many **servings** of fruit do you eat on **one** of those days? | | | | Number of servings  Don't Know 77 | | | | | | └─┴─┘ | | | | D2 |
| In a typical week, on how many days do you **eat vegetables**? | | | | Number of days Don't Know 77 | | | | | | *└─┴─┘ If 0 days, go to D5* | | | | D3 |
| How many **servings** of vegetables do you eat on **one** of those days? | | | | Number of servings  Don’t know 77 | | | | | | └─┴─┘ | | | | D4 |
| How often do you **add animal fats** to your food right before you eat it or as you are eating it? | | | | Always  Sometimes  Rarely  Never | | | | | | 1  2  3  4 | | | | D5 |
| How often do you **add salt** to your food right before you eat it or as you are eating it? | | | | Always | | | | | | | 1 | | | D6 |
|  |  |  |  | Sometimes | | | | | | | 2 | | |  |
|  |  |  |  | Rarely | | | | | | | 3 | | |  |
|  |  |  |  | Never | | | | | | | 4 | | |  |

| Do you consume red meat (beef, lamb, pork)? | Yes  No | | | | | 1  2 *If No, go to D10* | D7 |
| --- | --- | --- | --- | --- | --- | --- | --- |
| How many days do you consume red meat per week? | Number of days | | | | | └─┘ | D8 |
| How many times do you consume red meat in one of those days? | Number of times | | | | | └─┴─┘ | D9 |
| What is your typical portion size when you eat red meat? | Weight in grams/meal | | | | | └─┴─┴─┴─┘ | D10 |
| Do you ingest animal’s blood? | Yes  No | | | | | 1  2 *If No, go to P1* | D11 |
| What kind (fresh, processed) of blood do you ingest? | Fresh  Processed | | | | | 1  2 | D12 |
| How often do you ingest animal’s blood? | Occasionally  Often  Very often  Always | | | | | 1  2  3  4 | D13 |
| **Physical Activity** | | | | | | | |
| *Next, I am going to ask you about the time you spend doing different types of physical activity in a typical week. Please answer these questions even if you do not consider yourself to be a physically active person.* | | | | | | | |
| **Question** | **Response** | | | | | | **Code** |
| **Work** | | | | | | | |
| Does your work involve intensity activity that causes large increases in breathing or heart rate like *[carrying or lifting* *heavy loads, digging or construction work, herding animals]* for at least 10 minutes continuously? | Yes | | 1 | | | | P1 |
|  | No | | 2  *If No, go to P4* | | | |  |
| In a typical week, on how many days do you do intensity activities as part of your work? | Number of days | | └─┘ | | | | P2 |
| How much time do you spend doing vigorous-intensity activities at work on a typical day? | Hours: minutes | | └─┴─┘: └─┴─┘  hrs mins | | | | P3 (a-b) |
| **Travel to and from places** | | | | | | | |
| Do you walk or use a bicycle *(pedal cycle)* for at least 10 minutes continuously to get to and from places? | Yes | | 1 | | | | P4 |
|  | No | | 2  *If No, go to P7* | | | |  |
| In a typical week, on how many days do you walk or bicycle for at least 10 minutes continuously to get to and from places? | Number of days | | └─┘ | | | | P5 |
| How much time do you spend walking or bicycling for travel on a typical day? | Hours: minutes | | └─┴─┘: └─┴─┘  hrs mins | | | | P6 (a-b) |
| **Recreational activities** | | | | | | | |
| Do you do any intensity sports, fitness or recreational *(leisure)* activities that cause large increases in breathing or heart rate like *[running or football]* for at least 10 minutes continuously? | Yes | | 1 | | | | P7 |
|  | No | | 2  *If No, go to H1* | | | |  |
| In a typical week, on how many days do you do intensity sports, fitness or recreational *(leisure)* activities? | Number of days | | └─┘ | | | | P8 |
| How much time do you spend doing intensity sports, fitness or recreational activities on a typical day? | Hours: minutes | | └─┴─┘: └─┴─┘  hrs mins | | | | P9  (a-b) |
| **Sedentary behavior** | | | | | | | |
| *The following question is about sitting or reclining at work, at home, getting to and from places, or with friends including time spent sitting at a desk, sitting with friends, traveling in car, bus, reading, playing cards or watching television, but do not include time spent sleeping* | | | | | | | |
| How much time do you usually spend sitting or reclining on a typical day? | Hours: minutes | | └─┴─┘: └─┴─┘  hrs mins | | | | P10  (a-b) |
| **History of Raised Blood Pressure** | | | | | | | |
| Have you ever had your blood pressure measured by a doctor or other health worker? | Yes | | | 1 | | | H1 |
|  | No | | | 2 *If No, go to H3* | | |  |
| Have you ever been told by a doctor or other health worker that you have raised blood pressure or hypertension? | Yes | | | 1 | | | H2a |
|  | No | | | 2 *If No, go to H6* | | |  |
| Were you first told in the past 12 months? | Yes | | | 1 | | | H2b |
|  | No | | | 2 | | |  |
| In the past two weeks, have you taken any drugs (medication) for raised blood pressure prescribed by a doctor or other health worker? | Yes | | | 1 | | | H3 |
|  | No | | | 2 | | |  |
| Have you ever seen a traditional healer for raised blood pressure or hypertension? | Yes | | | 1 | | | H4 |
|  | No | | | 2 | | |  |
| Are you currently taking any herbal or traditional remedy for your raised blood pressure? | Yes | | | 1 | | | H5 |
|  | No | | | 2 | | |  |
| Is there anyone from you family (grandparents or parents) who has ever had raised blood pressure or hypertension? | Yes  No | | | 1  2 | | | H6 |
| **History of Diabetes** | | | | | | | |
| Have you ever had your blood sugar measured by a doctor or other health worker? | Yes | | | 1 | | | H7 |
|  | No | | | 2 *If No, go to H9* | | |  |
| Have you ever been told by a doctor or other health worker that you have raised blood sugar or diabetes? | Yes | | | 1 | | | H8a |
|  | No | | | 2  *If No, go to H14* | | |  |
| Were you first told in the past 12 months? | Yes | | | 1 | | | H8b |
|  | No | | | 2 | | |  |
| In the past two weeks, have you taken any drugs (medication) for diabetes prescribed by a doctor or other health worker? | Yes | | | 1 | | | H9 |
|  | No | | | 2 | | |  |
| Are you currently taking insulin for diabetes prescribed by a doctor or other health worker? | Yes | | | 1 | | | H10 |
|  | No | | | 2 | | |  |
| Have you ever seen a traditional healer for diabetes or raised blood sugar? | Yes | | | 1 | | | H11 |
|  | No | | | 2 | | |  |
| Are you currently taking any herbal or traditional remedy for your diabetes? | Yes | | | 1 | | | H12 |
|  | No | | | 2 | | |  |
| Is there anyone from you family (grandparents or parents) who has ever had diabetes? | Yes  No | | | 1  2 | | | H13 |
| **History of Raised Total Cholesterol** | | | | | | | |
| Have you ever had your cholesterol (fat levels in your blood) measured by a doctor or other health worker? | Yes | | | | 1 | | H14 |
|  | No | | | | 2 *If No, go to H16* | |  |
| Have you ever been told by a doctor or other health worker that you have raised cholesterol? | Yes | | | | 1 | | H15a |
|  | No | | | | 2 *If No, go to H20* | |  |
| Were you first told in the past 12 months? | Yes | | | | 1 | | H15b |
|  | No | | | | 2 | |  |
| In the past two weeks, have you taken any oral treatment (medication) for raised total cholesterol prescribed by a doctor or other health worker? | Yes | | | | 1 | | H16 |
|  | No | | | | 2 | |  |
| Have you ever seen a traditional healer for raised cholesterol? | Yes | | | | 1 | | H17 |
|  | No | | | | 2 | |  |
| Are you currently taking any herbal or traditional remedy for your raised cholesterol? | Yes | | | | 1 | | H18 |
|  | No | | | | 2 | |  |
| Is there anyone from you family (grandparents or parents) who has ever had cholesterol? | Yes  No | | | | 1  2 | | H19 |
| **History of Cardiovascular Diseases** | | | | | | | |
| Have you ever had a heart attack or chest pain from heart disease (angina) or a stroke (cerebrovascular accident or incident)? | Yes | 1 | | | | | H20 |
|  | No | 2 *If No, go to M1a* | | | | |  |
| Are you currently taking aspirin regularly to prevent or treat heart disease? | Yes | 1 | | | | | H21 |
|  | No | 2 | | | | |  |
| Are you currently taking statins (Lovastatin/Simvastatin/Atorvastatin or any other statin) regularly to prevent or treat heart disease? | Yes | 1 | | | | | H22 |
|  | Yes  No | 1  2 | | | | |  |
| Is there anyone from you family (grandparents or parents) who has ever had Cardiovascular diseases? | Yes  No | 1  2 | | | | | H23 |
| **Step 2 Physical Measurements** | | | | | | | |
| **Blood Pressure** | | | | | | | |
| **Question** | **Response** | | | | | | **Code** |
| Reading 1 | Systolic (mmHg) | | | └─┴─┴─┘ | | | M1a |
|  | Diastolic (mmHg) | | | └─┴─┴─┘ | | | M1b |
| Reading 2 | Systolic (mmHg) | | | └─┴─┴─┘ | | | M2a |
|  | Diastolic (mmHg) | | | └─┴─┴─┘ | | | M2b |
| Reading 3 | Systolic (mmHg) | | | └─┴─┴─┘ | | | M3a |
|  | Diastolic (mmHg) | | | └─┴─┴─┘ | | | M3b |
| **Height and Weight** | | | | | | | |
| **For women:** Are you pregnant? | Yes | | | 1 | | | M4 |
|  | No | | | 2 | | |  |
| Height | in Centimetres (cm) | | | └─┴─┴─┘. └─┘ | | | M5 |
| Weight | in Kilograms (kg) | | | └─┴─┴─┘.└─┘ | | | M6 |
| **Waist and Hip** | | | | | | | |
| Waist circumference | in Centimetres (cm) | | | └─┴─┴─┘. └─┘ | | | M7 |
| Hip circumference | in Centimetres (cm) | | | └─┴─┴─┘. └─┘ | | | M8 |
| **Step 3 Biochemical Measurements** | | | | | | | |
| **Blood Glucose** | | | | | | | |
| **Question** | **Response** | | | | | | **Code** |
| During the past 8 hours have you had anything to eat or drink, other than water? | Yes | | | 1 *If Yes, go to B3* | | | B1 |
|  | No | | | 2 *If No, go to B4* | | |  |
| Time of day blood specimen taken (24-hour clock) | Hour: minutes | | | └─┴─┘: └─┴─┘  hrs. mins | | | B2 |
| Random blood glucose  *[CHOOSE ACCORDINGLY: MMOL/L* ***OR*** *MG/DL]* | mmol/l | | | └─┴─┘. └─┴─┘ | | | B3 |
|  | mg/dl | | | └─┴─┴─┘.└─┘ | | |  |
| Fasting blood glucose  *[CHOOSE ACCORDINGLY: MMOL/L* ***OR*** *MG/DL]* | mmol/l | | | └─┴─┘. └─┴─┘ | | | B4 |
|  | mg/dl | | | └─┴─┴─┘.└─┘ | | |  |
| **Blood Lipids** | | | | | | | |
| Total cholesterol  *[CHOOSE ACCORDINGLY: MMOL/L* ***OR*** *MG/DL]* | mmol/l | | | └─┴─┘. └─┴─┘ | | | B5 |
|  | mg/dl | | | └─┴─┴─┘.└─┘ | | |  |
| **Triglycerides and HDL Cholesterol** | | | | | | | |
| Triglycerides  *[CHOOSE ACCORDINGLY: MMOL/L* ***OR*** *MG/DL]* | mmol/l | | | └─┴─┘. └─┴─┘ | | | B6 |
|  | mg/dl | | | └─┴─┴─┘.└─┘ | | |  |
| HDL Cholesterol  *[CHOOSE ACCORDINGLY: MMOL/L* ***OR*** *MG/DL]* | mmol/l | | | └─┘. └─┴─┘ | | | B7 |
|  | mg/dl | | | └─┴─┴─┘.└─┘ | | |  |

***(Thanks, participant, for his/her time and end the interview)***

***END***
