# Supplementary material for: Red meat consumption and its association with hypertension and hyperlipidaemia among adult Maasai pastoralists of Ngorongoro Conservation Area, Tanzania
Source: PLoS One. 2020 Jun 1;15(6):e0233777. doi: 10.1371/journal.pone.0233777 (PMC7263614; doi:10.1371/journal.pone.0233777)
Supplement: S2 File — (DOCX) [file pone.0233777.s002.docx]

## Swahili Questionnaire

Hili ni dodoso lililoandaliwa kutokana na dodoso la Shirika la Afya Duniani STEPS kuhusu magonjwa yasioambukizwa na kuongezewa baadhi ya maswali.

| **Taarifa za Utafiti** | | | | | | | | | | | | |
| --- | --- | --- | --- | --- | --- | --- | --- | --- | --- | --- | --- | --- |
| **Eneo na Tarehe** | | | | **Jibu** | | | | | | | | **Geresho** |
| Namba ya Eneo/Kijiji | | | | **└─┴─┴─┴─┴─┴─┘** | | | | | | | | I1 |
| Jina la Eneo/Kijiji | | | |  | | | | | | | | I2 |
| Namba ya Mhojiwa | | | | └─┴─┴─┴─┘ | | | | | | | | I3 |
| Tarehe ya mahojiano | | | | └─┴─┘ └─┴─┘ └─┴─┴─┴─┘  Siku mwezi mwaka | | | | | | | | I4 |
| **Ridhaa, Lugha ya mahojiano na Jina** | | | | **Jibu** | | | | | | | | **Geresho** |
| Je, umeelewa na kupata fomu ya ridhaa? | | | | Ndiyo | | | 1 | | | | | I5 |
|  |  |  |  | Hapana | | | 2 ***KAMA HAPANA, SITISHA MAHOJIANO*** | | | | |  |
| Lugha ya mahojiano | | | | Kiswahili | | | 1 | | | | | I6 |
|  |  |  |  | Kimasai | | | 2 | | | | |  |
|  |  |  |  |  | | |  | | | | |  |
| Muda wa Mahojiano  (nakili katika masaa 24) | | | | └─┴─┘: └─┴─┘  Saa Dakika | | | | | | | | I7 |
| **Taarifa za mhojiwa** | | | | | | | | | | | | |
| **Taarifa za Mhojiwa** | | | | | | | | | | | | |
| **Swali** | | **Jibu** | | | | | | | | | **Geresho** | |
| Jinsia(*gundua kwa kumtizama*) | | Mwanaume | | | | | | | 1 | | C1 | |
|  |  | Mwanamke | | | | | | | 2 | |  |  |
| Tarehe ya kuzaliwa  (*Kama hajui) 77 77 7777* | | └─┴─┘ └─┴─┘ └─┴─┴─┴─┘  Tarehe Mwezi Mwaka | | | | | | | | | C2 | |
| Una umri gani? | | Miaka | | | | | | | └─┴─┘ | | C3 | |
| Nini **kiwango chako cha elimu** ulichofikia? | | | Hakusoma | | | | | 1 | | | C4 | |
|  |  |  | Amemaliza elimu ya Msingi | | | | | 2 | | |  |  |
|  |  |  | Amemaliza elimu ya Sekondari | | | | | 3 | | |  |  |
|  |  |  | Amemaliza elimu ya juu | | | | | 4 | | |  |  |
| Hali yako ya ndoa | | | Sijaolewa | | | | | 1 | | | C5 | |
|  |  |  | Nimeolewa/Nimeoa | | | | | 2 | | |  |  |
|  |  |  | Mtalaka/Mjane | | | | | 3 | | |  |  |
| Katika kipindi cha miezi 12 iliyopita, shughuli yako kuu ilikuwa nini? | | | Hana kazi/mwanafunzi | | | | | 1 | | | C6 | |
|  |  |  | Mfugaji  Mkulima | | | | | 2  3 | | |  |  |
|  |  |  | Mfanyabiashara | | | | | 4 | | |  |  |
|  |  |  | Muajiriwa | | | | | 5 | | |  |  |
| Ni wanakaya wangapi wenye umri zaidi ya miaka 18 ikijumuisha na wewe wanaishi kwenye kaya hii? | | | Idadi ya watu | | | | | └─┴─┘ | | | C7 | |
| Nini wastani wa kipato cha kaya kwa mwaka uliopita?(*ANDIKA SEHEMU MOJA TU, NA SI MAJIBU YOTE 3*) | | | Kwa wiki | | └─┴─┴─┴─┴─┴─┴─┘ *Nenda T1* | | | | | | C8a | |
|  |  |  | Kwa mwezi | | └─┴─┴─┴─┴─┴─┴─┘ *Nenda T1* | | | | | | C8b | |
|  |  |  | Kwa mwaka | | └─┴─┴─┴─┴─┴─┴─┘ *Nenda T1* | | | | | | C8c | |
|  |  |  | Hafahamu | | 88 └─┴─┘ | | | | | | C8d | |
| Hatua ya 1 **Mtindo wa maisha** | | | | | | | | | | | | |
| **Matumizi ya Tumbaku** | | | | | | | | | | | | |
| *Sasa naenda kukuuliza maswali kuhusiana na matumizi ya Tumbaku* | | | | | | | | | | | | |
| **Swali** | **Majibu** | | | | | | | | | **Geresho** | | |
| Je, unavuta aina yoyote ya tumbaku; kama sigara,au sigara kubwa au msokoto wowote? | Ndiyo | | | | | 1 | | | | T1 | | |
|  | Hapana | | | | | 2 | | | |  |  |  |
| Je unanusa au kutafuna aina yoyote ya tumbaku? | Ndiyo  Hapana | | | | | 1  2 *Kama Hapana, nenda A1* | | | | T2 | | |
| Je, unavuta/ kunusa/kutafuna tumbaku au aina yoyote ya msokoto kila siku? | Ndiyo | | | | | 1 | | | | T3 | | |
|  | Hapana | | | | | 2 | | | |  |  |  |
| Ulianza kutumia tumbaku ukiwa na umri gani? | Umri (Miaka) | | | | | **└─┴─┘** | | | | T4 | | |
|  | weka 77 kama hajui | | | | |  |  |  |  |  |  |  |

| **Matumizi ya Pombe** | | | | | | | |
| --- | --- | --- | --- | --- | --- | --- | --- |
| *Maswali yafuatayo nitakuuliza maswali kuhusu matumizi ya Pombe* | | | | | | | |
| **Swali** | **Majibu** | | | | | | **Geresho** |
| Je, unatumia pombe aina yoyote ile kama bia, spiriti kama konyagi, mvinyo, pombe ya kienyeji unayotengeneza nyumbani au nchi nyingine? | Ndiyo | | 1 | | | | A1 |
|  | Hapana | | 2 | | | |  |
| Je, umekunywa pombe katika kipindi cha miezi 12 iliyopita? | Ndiyo | | 1 *Kama jibu ni 1; Nenda A4* | | | | A2 |
|  | Hapana | | 2 | | | |  |
| Je, umeacha kunywa pombe kutokana na sababu za kiafya, kama athari za pombe kwa afya yako;  Ushauri wa daktari au mhudumu wa afya? | Ndiyo | | 1 *Kama jibu ni 1; Nenda D1* | | | | A3 |
|  | Hapana | | 2 *Kama jibu ni 1; Nenda D1* | | | |  |
| Kwa kipindi cha miezi 12 iliyopita, ni mara ngapi umekua ukinywa pombe? | Kila siku | | 1 | | | | A4 |
|  | Siku 5-6 kwa wiki | | 2 | | | |  |
|  | Siku 3-4 kwa wiki | | 3 | | | |  |
|  | Siku1-2 kwa wiki  Siku 1-3 kwa mwezi  Pungufu ya x1 kwa mwezi | | 4  5  6 | | | |  |
|  | Sijanywa kabisa | | 7 | | | |  |
| Umekunywa pombe ndani ya siku 30 zilizopita? | Ndio  Hapana | | 1  2 ***Kama 2, Nenda D1*** | | | | A5 |
| Ndani ya siku 7 zilizopita umekunywa pombe yoyote ya kienyejji? (kama Ng’otoroki?) | Ndio  Hapana | | 1  2 | | | | A6 |
| **Matumizi ya Chakula** | | | | | | | |
| *Katika kipengele hiki nitakuuliza juu ya matumizi ya matunda, mbogamboga, chumvi na nyama nyekundu.* | | | | | | | |
| **Swali** | | **Majibu** | | | | **Geresho** | |
| Katika wiki ya kawaida ni siku ngapi huwa unakula matunda? | | Idadi ya siku Hafahamu weka 77 | | └─┴─┘  *Kama 0; Nenda D3* | | D1 | |
| Ni milo mingapi ya matunda huwa unakula ndani ya siku hizo? | | Idadi ya Milo  Hafahamu weka 77 | | └─┴─┘ | | D2 | |
| Katika wiki ya kawaida ni siku ngapi huwa unakula mboga za majani? | | Idadi ya siku Hafahamu weka 77 | | └─┴─┘  *Kama 0; Nenda D5* | | D3 | |
| Ni milo mingapi ya mboga za majani huwa unakula ndani ya siku hizo? | | Idadi ya Milo  Hafahamu weka 77 | | └─┴─┘ | | D4 | |
| Ni mara ngapi huwa unaongeza mafuta ya wanyama kwenye chakula chako wakati unakula? | | Mara kwa mara  Mara nyingine  Mara chache  Kamwe sijatumia | | 1  2  3  4 | | D5 | |
| Ni mara ngapi huwa unaongeza chumvi kwenye chakula chako wakati unakula? | | Mara kwa mara | | | 1 | D6 | |
|  |  | Mara nyingine | | | 2 |  |  |
|  |  | Mara chache | | | 3 |  |  |
|  |  | Kamwe sijatumia | | | 4 |  |  |

| Je, unatumia nyama nyekundu (nyama ya ng’ombe,kondoo na nguruwe)? | Ndiyo  Hapana | 1  2  *Hapana, Nenda D10* | D7 |
| --- | --- | --- | --- |
| Ni siku ngapi ndani ya wiki unatumia nyama nyekundu? | Idadi ya siku | └─┘ | D8 |
| Ni mara ngapi unakula nyama nyekundu ndani ya siku hizo? | Idadi ya mara | └─┴─┘ | D9 |
| Kwa mlo mmoja ni kiasi gani cha nyama huwa unatumia? | Uzito kwa gramu/mlo | └─┴─┴─┴─┘ | D10 |
| Unatumia/kunywa damu kutoka kwa wanyama? | Ndio  Hapana | 1  2  *Hapana, Nenda P1* | D11 |
| Ni aina gani(freshi au iliyopikwa) ya damu huwa unakunywa? | Freshi  Iliyopikwa  Zote | 1  2  3 | D12 |
| Unaweza kusema ni mara ngapi huwa unakunywa damu? | Mara chache  Mara kwa mara  Mara nyingi  Kila mara | 1  2  3  4 | D13 |

| **Shughuli zinazo hangaisha mwili** | | | | | | | | | | | |
| --- | --- | --- | --- | --- | --- | --- | --- | --- | --- | --- | --- |
| *Katika kipengele hiki, nitakuuliza kuhusu shughuli ulizofanya kwa kipindi cha wiki nzima. Tafadhali nijibu hata kama wewe binafsi hauzioni kama ni shughuli za kuhangaisha mwili* | | | | | | | | | | | |
| **Swali** | | **Majibu** | | | | | | | **Geresho** | | |
| **Kazi** | | | | | | | | | | | |
| Je, kazi yako inahusisha shuguli ambazo hukusababishia mapigo ya moyo kwenda mbio au kupumua kwa haraka angalau dakika 10 mfululizo? (*Mfano; kubeba vitu vizito, kulima au kujenga na kuswaga wanyama*) | | Ndiyo 1 | | | | | |  | P1 | | |
|  |  | Hapana 2 | | | | | | *Kama Hapana, Nenda P4* |  |  |  |
| Katika wiki ya kawaida ni siku ngapi hufanya shuguli za kutumia nguvu kama sehemu ya kazi zako? | | Idadi ya siku | | | | | | └─┘ | P2 | | |
| Ni muda gani huwa unatumia kufanya shughuli za kutumia nguvu kwa siku? | | Muda | | | | | | └─┴─┘: └─┴─┘  Saa dakika | P3 (a-b) | | |
| **Kusafiri kutoka sehemu moja kwenda jingine** | | | | | | | | | | | |
| Je, unatembea kwa mguu au kutumia baiskeli kwenda na kurudi sehemu fulani kwa angalau dakika 10? | | Ndiyo | | | | | | 1 | P4 | | |
|  |  | Hapana | | | | | | 2 *Kama Hapana, nenda P7* |  |  |  |
| Ni siku ngapi kwa wiki ulitembea au kuendesha baiskeli kwenda na kurudi eneo fulani kwa angalau dakika 10? | | Idadi ya siku | | | | | | └─┘ | P5 | | |
| Je, huwa unatumia muda gani kutembea au kuendesha baiskeli kwa siku nzima? | | Muda | | | | | | └─┴─┘: └─┴─┘  Saa dakika | P6 (a-b) | | |
| **Burudani na mazoezi** | | | | | | | | | | | |
| Je, unafanya mazoezi *(burudani*) yanayosababisha kuongezeka kwa mapigo ya moyo au kupumua kwa angalau dakika 10 mfululizo? (*Mfano; kukimbia au kucheza mpira*) | | Ndio 1 | | | | | |  | P7 | | |
|  |  | Hapana 2 | | | | | | *Kama Hapana, nenda H1* |  |  |  |
| Ni siku ngapi za wiki huwa unafanya mazoezi ya viungo au michezo ya burudani? | | Idadi ya siku | | | | | | └─┘ | P8 | | |
| Huwa unatumia muda gani kufanya mazoezi ya viungo au michezo ya burudani kwa siku? | | Muda | | | | | | └─┴─┘: └─┴─┘  Saa dakika | P9  (a-b) | | |
| Katika siku ya kawaida, ni muda kiasi gani unatumia kukaa au kuketi? | | Muda | | | | | | └─┴─┘: └─┴─┘  Saa dakika | P10  (a-b) | | |
| **Historia ya shinikizo la damu** | | | | | | | | | | | |
| Je, uliwahi kupimwa shinikizo la damu na daktari au mhudumu wa afya? | | Ndio 1 | | | | |  | | H1 | | |
|  |  | Hapana 2 | | | | | *Kama Hapana, nenda H3* | |  |  |  |
| Je, uliwahi kuambiwa na daktari au mhudumu wa afya kuwa una tatizo la shinikizo la damu?  Je, uliambiwa ndani ya miezi 12 iliyopita? | | Ndio | | | | | 1 | | H2a  H2b | | |
|  |  | Hapana | | | | | 2 *Kama Hapana, nenda H6* | |  |  |  |
|  |  | Ndio  Hapana | | | | | 1  2 | |  |  |  |
|  |  |  | | | | |  | |  |  |  |
| Katika wiki 2 zilizopita, umetumia dawa yoyote kwa ajili ya shinikizo la damu uliopewa na daktari au mhudumu wa afya? | | Ndio  Hapana | | | | | 1  2 | | H3 | | |
| Umewahi kumuona mtaalamu wa tiba za asili kwa ajili ya shinikizo la damu | | Ndio  Hapana | | | | | 1  2 | | H4 | | |
| Kwa sasa, unatumia dawa yoyote ya kienyeji kwa ajili ya kutibu shinikizo la damu? | | Ndio  Hapana | | | | | 1  2 | | H5 | | |
| Je, kuna ndugu yako wa karibu (bibi/babu/baba/mama) aliyewahi kuwa na tatizo la shinikizo la damu? | | Ndio  Hapana | | | | | 1  2 | | H6 | | |
| **Historia ya kisukari** | | | | | | | | | | | |
| Je, uliwahi kupimwa ugonjwa wa Kisukari na daktari au mhudumu wa afya? | | Ndio | | | | | 1 | | H7 | | |
|  |  | Hapana | | | | | 2 *Kama Hapana, nenda H9* | |  |  |  |
| Je, uliwahi kuambiwa na daktari au mhudumu wa afya kuwa una tatizo la Kisukari? | | Ndio | | | | | 1 | | H8a | | |
|  |  | Hapana | | | | | 2 *Kama Hapana, nenda H13* | |  |  |  |
| Je, uliambiwa ndani ya miezi 12 iliyopita? | | Ndio  Hapana | | | | | 1  2 | | H8b | | |
| Katika wiki 2 zilizopita, umetumia dawa yoyote kwa ajili ya Kisukari uliopewa na daktari au mhudumu wa afya? | | Ndio  Hapana | | | | | 1  2 | | H9 | | |
| Kwa sasa unatumia insulini kwaajili ya Kisukari uliopewa na dactari au mhudumu wa afya? | | Ndio  Hapana | | | | | 1  2 | | H10 | | |
| Umewahi kumuona mtaalamu wa tiba za asili kwa ajili ya matibabu ya ongezeko la sukari kwenye damu? | | Ndio  Hapana | | | | | 1  2 | | H11 | | |
| Kwa sasa, unatumia dawa yoyote ya kienyeji kwa ajili ya Kisukari? | | Ndio  Hapana | | | | | 1  2 | | H12 | | |
| Je kuna ndugu yako wa karibu (bibi/babu/mama/baba) aliyewahi kuwa na tatizo la kisukari? | | Ndio  Hapana | | | | | 1  2 | | H13 | | |
| **Historia ya kiasi cha Lehemu mwilini** | | | | | | | | | | | |
| Je, uliwahi kupimwa na daktari au mhudumu wa afya tatizo la kuongezeka kwa mafuta/lehemu kwenye damu? | | Ndiyo | | | 1 | | | | | H14 |  |
|  |  | Hapana | | | 2 *Kama Hapana, nenda H16* | | | | |  |  |
| Je, uliwahi kuambiwa na daktari au mhudumu wa afya kuwa una tatizo la kuongezeka kwa mafuta/lehemu kwenye damu? | | Ndiyo | | | 1 | | | | | H15a |  |
|  |  | Hapana | | | 2 *Kama Hapana, nenda H19* | | | | |  |  |
| Je, uliambiwa ndani ya miezi 12 iliyopita? | | Ndio  Hapana | | | 1  2 | | | | | H15b |  |
| Katika wiki 2 zilizopita, umemeza dawa yoyote kwa ajili ya matibabu ya kuongezeka kwa mafuta/lehemu kwenye damu uliopewa na daktari au mhudumu wa afya? | | Ndio  Hapana | | | 1  2 | | | | | H16 |  |
| Umewahi kumuona mtaalamu wa tiba za asili kwa ajili ya kuongezeka kwa mafuta/lehemu kwenye damu? | | Ndio  Hapana | | | 1  2 | | | | | H17 |  |
| Kwa sasa, unatumia dawa yoyote ya kienyeji kwa ajili ya kuongezeka kwa mafuta/lehemu kwenye damu? | | Ndio  Hapana | | | 1  2 | | | | | H18 |  |
| Je kuna ndugu yako wa karibu (bibi/babu/mama/baba) aliyewahi kuwa na tatizo la lehemu mwilini? | | Ndio  Hapana | | | 1  2 | | | | | H19 |  |
| **Historia ya Magonjwa ya Moyo** | | | | | | | | | | | |
| Je, uliwahi kupatwa na tatizo la shambulio la Moyo, maumivu makali chini ya kifua au kupooza? | | | | Ndiyo | 1 | | | | | H20 | |
|  |  |  |  | Hapana | 2 *Kama Hapana, nenda M1a* | | | | |  |  |
| Je, unatumia Asprini kila mara kujikinga au kutibu matatizo ya Moyo? | | | | Ndiyo | 1 | | | | | H21 | |
|  |  |  |  | Hapana | 2 | | | | |  |  |
| Je, unatumia dawa zozote za hospitali kuzuia au kutibu matatizo ya moyo mara kwa mara? | | | | Ndiyo | 1 | | | | | H22 | |
|  |  |  |  | Hapana | 2 | | | | |  |  |
| Je kuna ndugu yako wa karibu (bibi/babu/mama/baba) aliyewahi kuwa na tatizo la ugonjwa wa moyo? | | | | Ndio  Hapana | 1  2 | | | | | H23 | |
| **Hatua ya 2 Vipimo vya Mwili** | | | | | | | | | | | |
| **Shinikizo la Damu** | | | | | | | | | | | |
| **Swali** | | **Majibu** | | | | | | | **Geresho** | | |
| Mara ya kwanza | | Presha ya juu (mmHg) | | | | | └─┴─┴─┘ | | M1a | | |
|  |  | Presha ya chini (mmHg) | | | | | └─┴─┴─┘ | | M1b | | |
| Mara ya pili | | Presha ya juu (mmHg) | | | | | └─┴─┴─┘ | | M2a | | |
|  |  | Presha ya chini (mmHg) | | | | | └─┴─┴─┘ | | M2b | | |
| Mara ya tatu | | Presha ya juu (mmHg) | | | | | └─┴─┴─┘ | | M3a | | |
|  |  | Presha ya chini(mmHg) | | | | | └─┴─┴─┘ | | M3b | | |
| **Vipimo vya Urefu na Uzito** | | | | | | | | | | | |
| **Kwa Wanawake tu**: Je, wewe ni mjamzito? | | Ndiyo 1 | | | | |  | | M4 | | |
|  |  | Hapana 2 | | | | |  | |  |  |  |
| Urefu | | Sentimita (cm) | | | | | └─┴─┴─┘. └─┘ | | M5 | | |
| Uzito | | Kilogram (kg) | | | | | └─┴─┴─┘.└─┘ | | M6 | | |
| Kiuno | | Sentimita (cm) | | | | | └─┴─┴─┘. └─┘ | | M7 | | |
| Hipsi | | Sentimita (cm) | | | | | └─┴─┴─┘. └─┘ | | M8 | | |
| **Hatua ya 3 Vipimo vya Biochemical** | | | | | | | | | | | |
| **Kiasi cha sukari kwenye damu** | | | | | | | | | | | |
| **Swali** | | **Jibu** | | | | | | | **Geresho** | | |
| Katika masaa 8 yaliyopita je, umekula chakula chochote tofauti na maji? | | Ndiyo | | | | | 1 *Kama Ndiyo, nenda B3* | | B1 | | |
|  |  | Hapana | | | | | 2 *Kama Hapana, nenda B4* | |  |  |  |
| Muda kipimo kilipochukuliwa | | Muda | | | | | └─┴─┘: └─┴─┘  Saa dakika | | B2 | | |
| Kiwango cha sukari kwenye damu (random)  [*CHAGUA KWA MFUMO HUU: MMOL/L* ***OR*** *MG/DL*] | | mmol/l | | | | | └─┴─┘. └─┴─┘ | | B3 | | |
|  |  | mg/dl | | | | | └─┴─┴─┘.└─┘ | |  |  |  |
| Kiwango cha sukari kwenye damu (fasting)  [*CHAGUA KWA MFUMO HUU: MMOL/L* ***OR*** *MG/DL*] | | mmol/l | | | | | └─┴─┘. └─┴─┘ | | B4 | | |
|  |  | mg/dl | | | | | └─┴─┴─┘.└─┘ | |  |  |  |
| **Chembe za mafuta kwenye damu [Blood Lipids]** | | | | | | | | | | | |
| Kiasi cha mafuta kwenye damu: *Total cholesterol*  [*CHAGUA KWA MFUMO HUU: MMOL/L* ***OR****MG/DL*] | | mmol/l | | | | | └─┴─┘. └─┴─┘ | | B5 | | |
|  |  | mg/dl | | | | | └─┴─┴─┘.└─┘ | |  |  |  |
| **Chembe za mafuta kwenye damu [Triglycerides and HDL Cholesterol]** | | | | | | | | | | | |
| Kiasi cha mafuta kwenye damu: *Triglycerides*  [*CHAGUA KWA MFUMO HUU: MMOL/L* ***OR*** *MG/DL*] | | mmol/l | | | | | └─┴─┘. └─┴─┘ | | B6 | | |
|  |  | mg/dl | | | | | └─┴─┴─┘.└─┘ | |  |  |  |
| Kiasi cha mafuta kwenye damu: *HDL Cholesterol*  *[CHAGUA KWA MFUMO HUU: MMOL/L* ***OR*** *MG/DL]* | | mmol/l | | | | | └─┘. └─┴─┘ | | B7 | | |
|  |  | mg/dl | | | | | └─┴─┴─┘.└─┘ | |  |  |  |

***(Mshukuru mshiriki wa muwa wake na ufunge mahojiano)***

***END***
