# Supplementary material for: Red meat consumption and its association with hypertension and hyperlipidaemia among adult Maasai pastoralists of Ngorongoro Conservation Area, Tanzania
Source: PLoS One. 2020 Jun 1;15(6):e0233777. doi: 10.1371/journal.pone.0233777 (PMC7263614; doi:10.1371/journal.pone.0233777)
Supplement: Attachment — Submitted filename: Response to Reviewers.docx [file pone.0233777.s003.docx]

**KILIMANJARO CHRISTIAN MEDICAL UNIVERSITY COLLEGE**

***(A constituent of College of Tumaini University Makumira)***

**P. O. Box 2240, MOSHI, Telephone: +255-027-2753616.**

**Tanzania. Fax: +255-27-2751351.**

**Email:** [**info@kcmuco.ac.tz**](mailto:info@kcmuco.ac.tz) **Web site:** [**http://www.kcmuco.ac.tz**](http://www.kcmuco.ac.tz)

**----------------------------------------------------------------------------------------------------------------------**

18^th^ March 2020

Venkata Naga Srikanth Garikipati
Academic Editor
PLOS ONE

**Dear Editor,**

**Re: Response to reviewers of “RED MEAT CONSUMPTION AND ITS ASSOCIATION WITH HYPERTENSION AND HYPERLIPIDEMIA AMONG ADULT MAASAI PASTORALISTS IN NGORONGORO CONSERVATION AREA, TANZANIA” manuscript.**

I sincerely thanks to all reviewer comments and their time to review our manuscript. Below are the responses regarding comments raised by both editor and reviewers.

**Editor Comments:**

**1. Please ensure that your manuscript meets PLOS ONE's style requirements, including those for file naming. The PLOS ONE style templates can be found at** <http://www.plosone.org/attachments/PLOSOne_formatting_sample_main_body.pdf> and <http://www.plosone.org/attachments/PLOSOne_formatting_sample_title_authors_affiliations.pdf>

**RESPONSE:** The manuscript followed the PLOS ONE’s style requirements.

**2. Please include additional information regarding the survey or questionnaire used in the study and ensure that you have provided sufficient details that others could replicate the analyses. For instance, if you developed a questionnaire as part of this study and it is not under a copyright more restrictive than CC-BY, please include a copy, in both the original language and English, as Supporting Information. Moreover, please include more details on how the questionnaire was pre-tested, and whether it was validated.**

**RESPONSE:** Data collection tool was adopted from WHO Non-Communicable Diseases Stepwise approach to surveillance (STEPS) <https://www.who.int/ncds/surveillance/steps/en/> This was then modified with additional questions to account for cultural diet of Maasai in Tanzania (attached in supporting information in both Swahili and English language). The questionnaire included; social demographic information (age gender, income etc), data on life style (physical activity, tobacco use, alcohol consumption, dietary intake) and relevant family history information was collected from each participant through self-reporting.Training of the data collection activity was conducted for five days. Pre-test of the tools and procedures were done in 4^th^ day of training to the community around Kilimanjaro Christian Medical University College area. After the pre-test, feedback was shared, challenges observed from the pre-test were explained and finalization of the tools were done.

**3. We note that you have stated that you will provide repository information for your data at acceptance. Should your manuscript be accepted for publication, we will hold it until you provide the relevant accession numbers or DOIs necessary to access your data. If you wish to make changes to your Data Availability statement, please describe these changes in your cover letter and we will update your Data Availability statement to reflect the information you provide.**

**RESPONSE:** No changes of the data availability statement.

**4.Please include captions for your Supporting Information files at the end of your manuscript, and update any in-text citations to match accordingly. Please see our Supporting Information guidelines for more information:** [**http://journals.plos.org/plosone/s/supporting-information**](http://journals.plos.org/plosone/s/supporting-information)**.**

**RESPONSE:** Captions of the supporting information are included in the manuscript.

**Reviewers' comments:**

**RESPONSE:** We thank reviewer 1 and 2 for positive comments regarding different sections of our manuscript, specifically with regard on question 1, 2, 3, 4 and 6 as there is no action needed. Below is our response to reviewer # 2 comments on question 5.

5. Review Comments to the Author

**Reviewer #2: The authors in this manuscript provide a population-based study of the Tanzanian Maasai community's red meat consumption. -Owing to the limitation in the socio-economic status of this community, their lifestyle governs a lot on their food habits. -Having excess red meat consumption leads to hyperlipidemia.**

**This work can help in providing people of that region an insight to develop a method of proper balance diet to combat cardiovascular diseases.

The authors could have tried to overcome the limitation of not defining the lipid profile in details, by use of a simple diagnostic kit used these days for biometric screening. However, I still would like to accept this manuscript for publication as this can help the developing countries raise their dietary standards along with WHO guidance.**

**RESPONSE:** We completely agree with the reviewer comment on these. However, due to the limited funding available during the conduct of this study we couldn’t explore much on lipids. We believe this study will provide data for further robust studies to explore in details the association of red meat and lipids as well as hypertension in this Masai community.

Kindly find our revised manuscript uploaded for further consideration.

Sincerely,

Ester Jacob Diarz

Corresponding author.
